# Supplementary material for: Chemical, Bioactivity, and Biosynthetic Screening of Epiphytic Fungus Zasmidium pseudotsugae
Source: Molecules. 2020 May 19;25(10):2358. doi: 10.3390/molecules25102358 (PMC7287617; doi:10.3390/molecules25102358)
Supplement: Supplementary file 1 [file molecules-25-02358-s001.pdf]

# Supplementary Information

# Chemical, bioactivity and biosynthetic screening of epiphytic fungus *Zasmidium pseudotsugae*

**Gisela A. González-Montiel [1], Elizabeth N. Kaweesa [1,*], Nicolas Feau [2], Richard C. Hamelin [2,3], Jeffrey K. Stone [4], and Sandra Loesgen [1,*,^]**

[1] Department of Chemistry, Oregon State University, Corvallis, Oregon 97331, USA; gonzalgi@oregonstate.edu (G.A.G-M), el.kaweesa@whitney.ufl.edu (E.N.K)
[2] Department of Forest and Conservation Sciences, University of British Columbia, Vancouver, British Columbia, Canada; nicolas.feau@ubc.edu
[3] Faculté de Foresterie et Géomatique, Institut de Biologie Intégrative et des Systèmes (IBIS), Université Laval, Québec, Canada; richard.hamelin@ubc.edu
[4] Department of Botany and Plant Pathology, Oregon State University, Corvallis, Oregon 97331, USA; stonej@science.oregonstat.edu

* Present address: Whitney Laboratory for Marine Bioscience and Department of Chemistry, University of Florida, St. Augustine, FL, USA
^ Author to whom correspondence should be addressed: sandra.loesgen@whitney.ufl.edu (S.L)

**Table of contents:**

LC/MS analysis of 8,8'-bijuglone (1) ……………………………………………………………2

HR/MS analysis of 8,8'-bijuglone (1) ……………………………………………………………3

NMR spectra of 8,8'-bijuglone (1) ……………………………………………………………4

IR spectrum of 8,8'-bijuglone (1) ……………………………………………………………...9

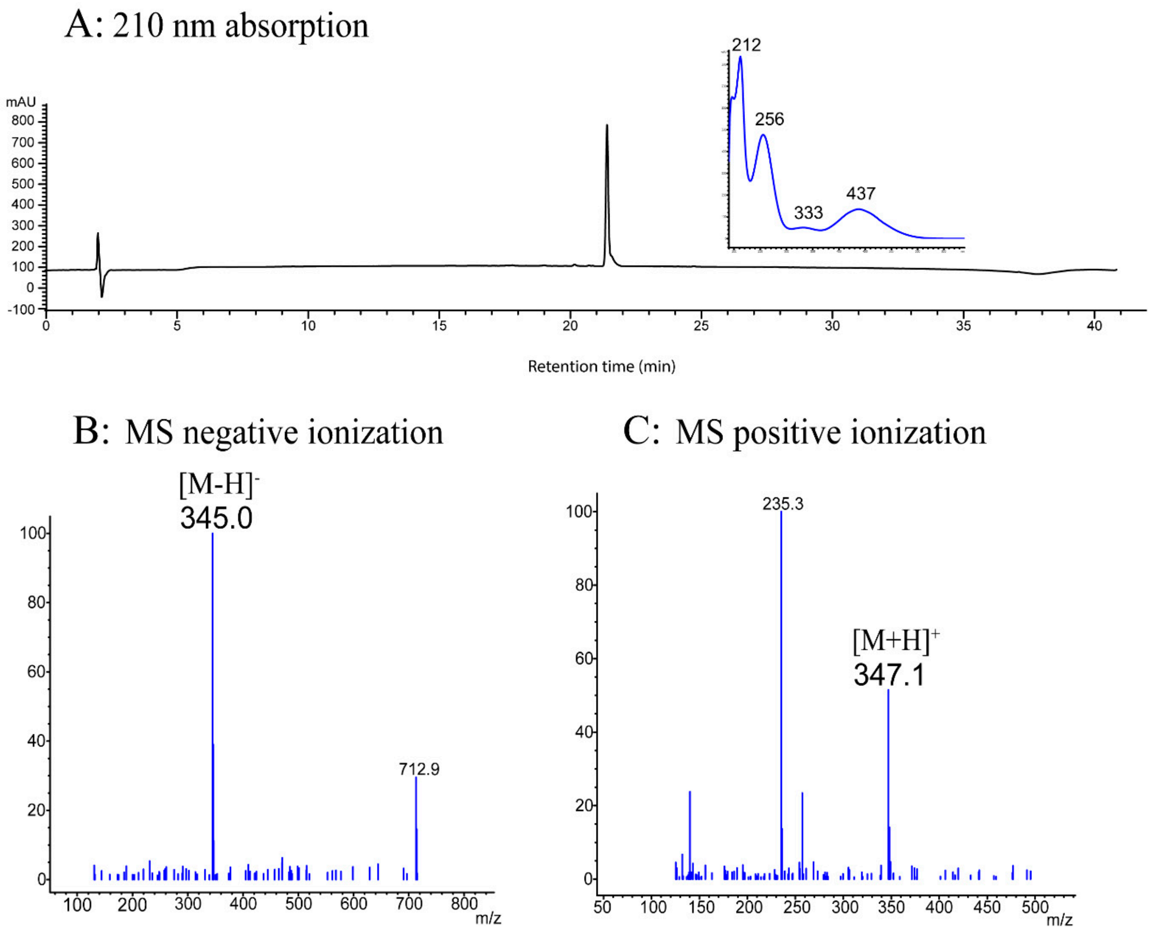


**Figure A1.** LC/MS analysis of pure 8,8'-bijuglone (**1**), C18 column, isocratic 50% ACN:50%water. (A) Absorption at 210 nm in black with UV chromophore insert in blue, (B) MS spectrum in negative ionization, (C) MS spectrum in positive ionization.

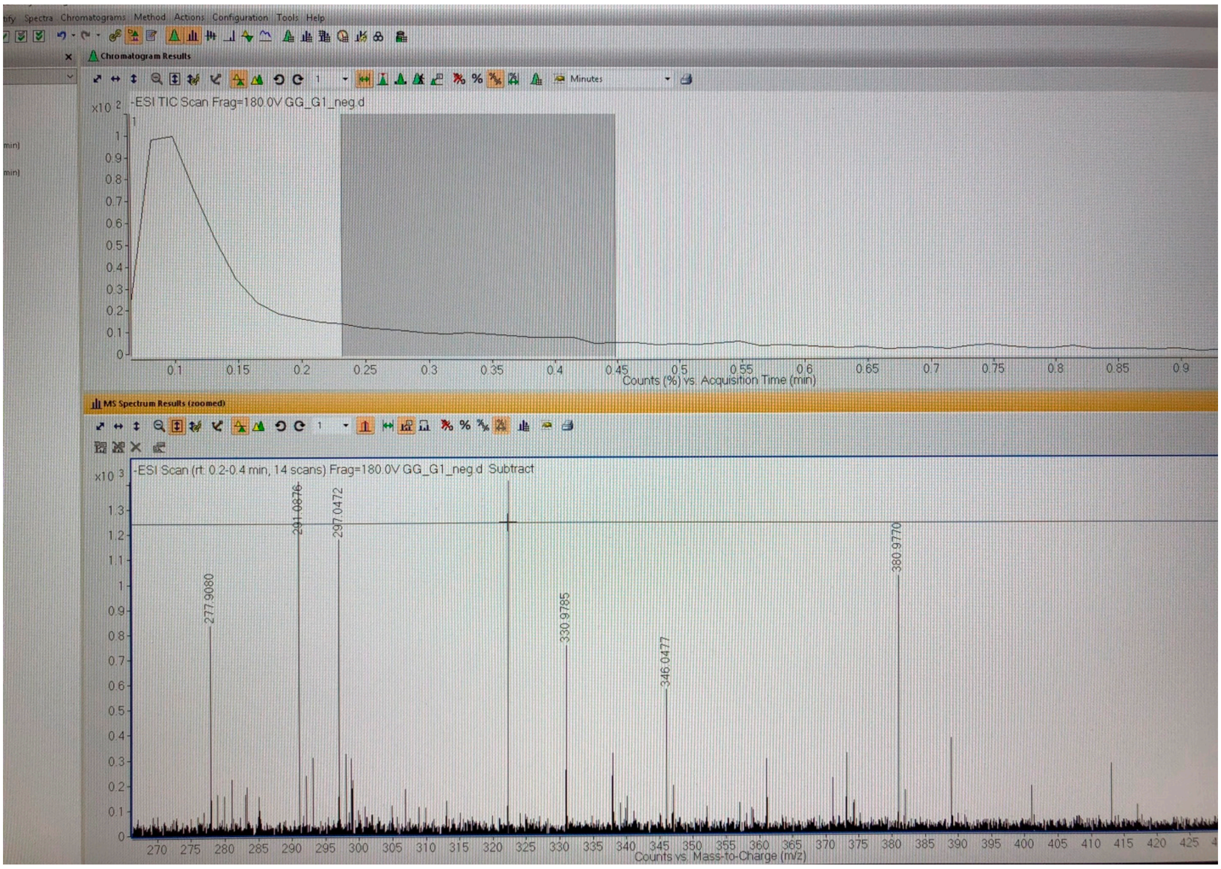


**Figure A2**. HR/MS analysis of pure 8,8'-bijuglone (1). Direct injection, negative mode, on QTOF-HRMS with *m/z* 346.0477 $[M]^-$ calcd. for $C_{20}H_{10}O_6^-$ 346.04829.

Apologies, due to the COVID-19 outbreak April/May 2020, we were not able to receive the original data file from the instrument to create a figure.

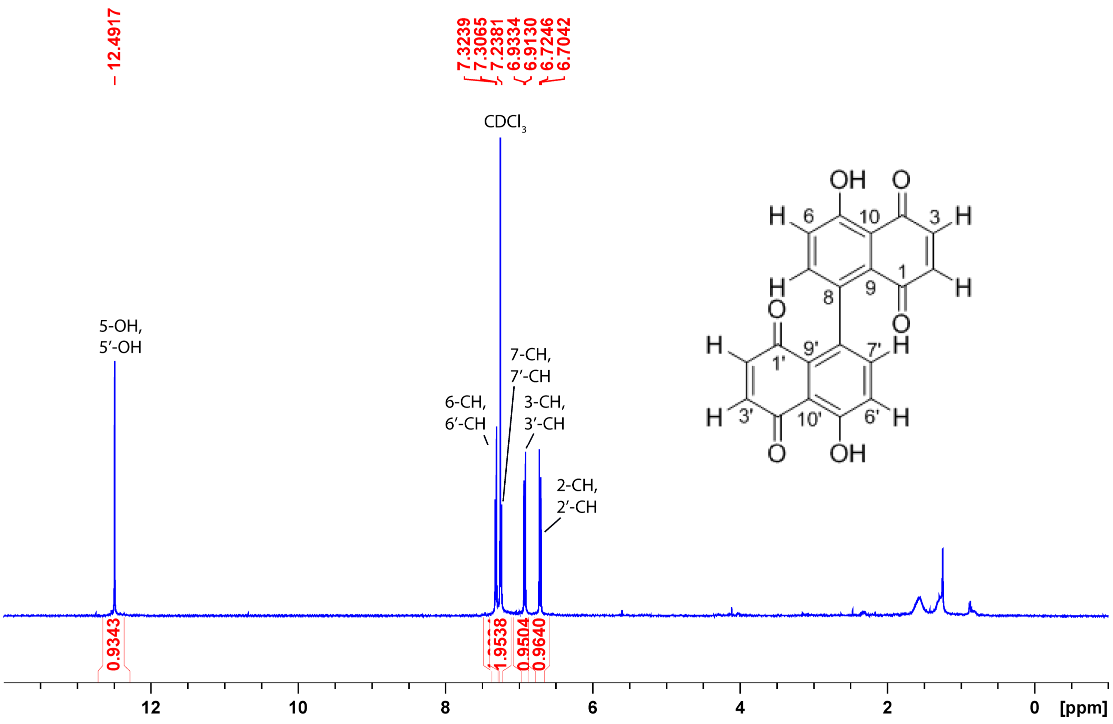


**Figure A3.** $^{1}$H-NMR spectrum of 8,8'-bijuglone (**1**) in $CDCl_3$ (500 MHz).

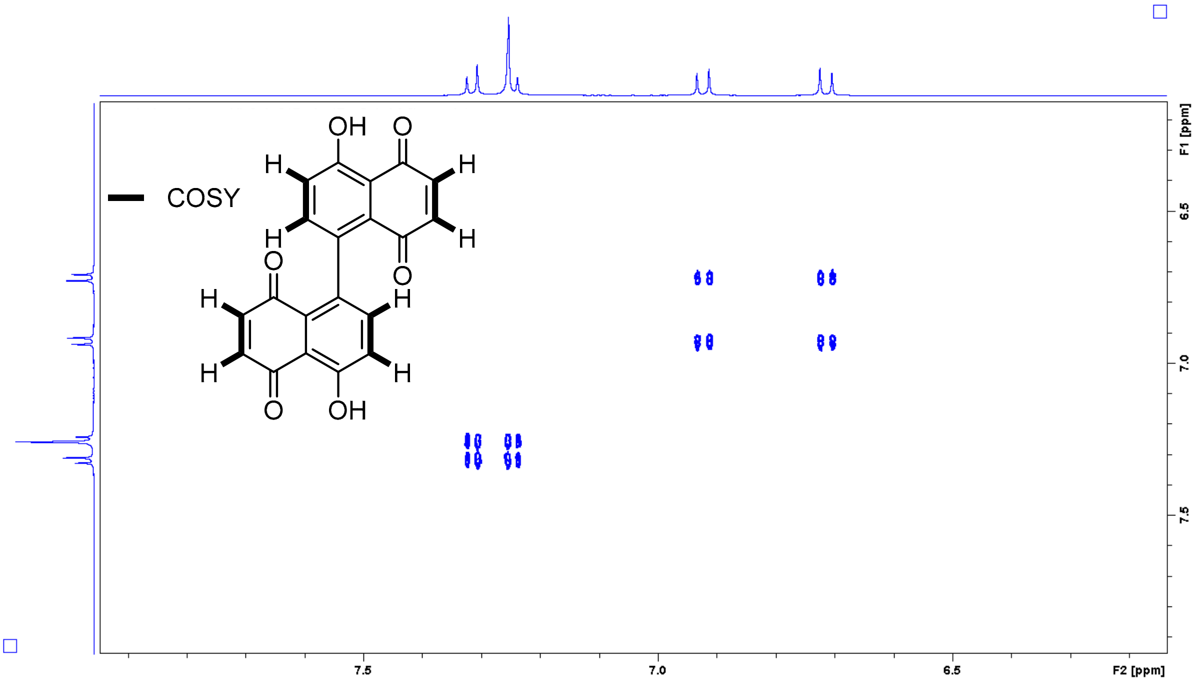


**Figure A4.** COSY spectrum of 8,8'-bijuglone (**1**) in $CDCl_3$ (500 MHz).

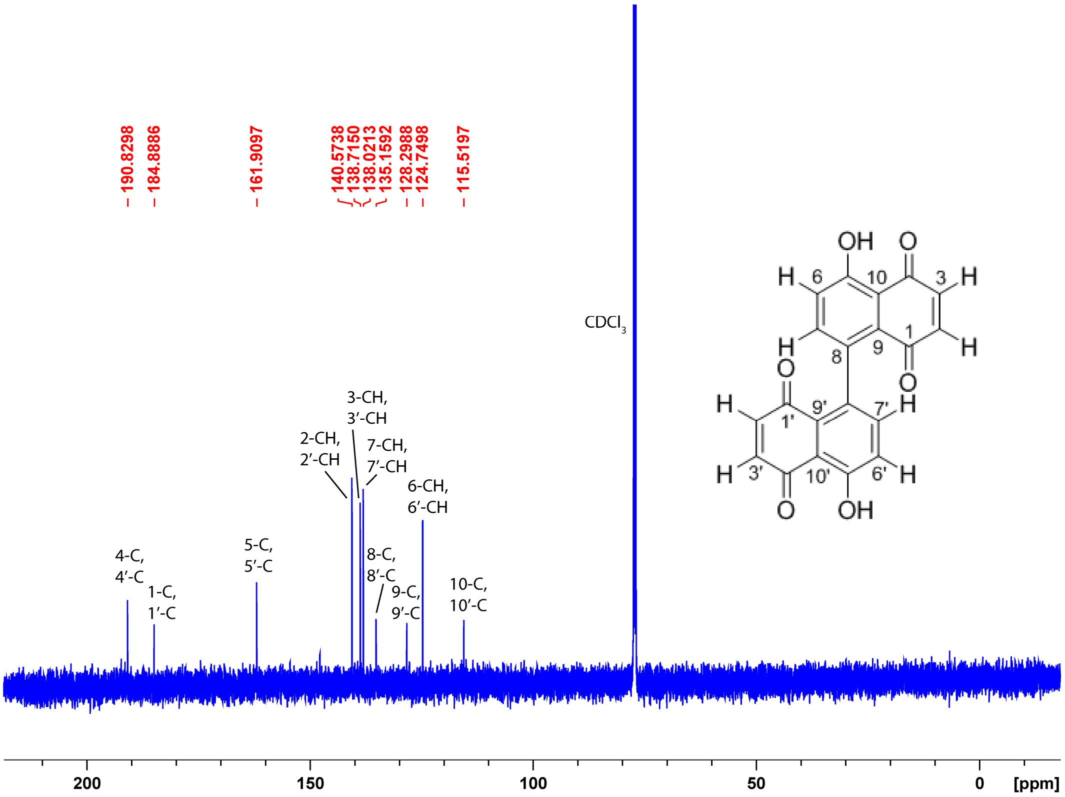


**Figure A5.** $^{13}$C-NMR spectrum of 8,8'-bijuglone (**1**) in $CDCl_3$ (500 MHz).

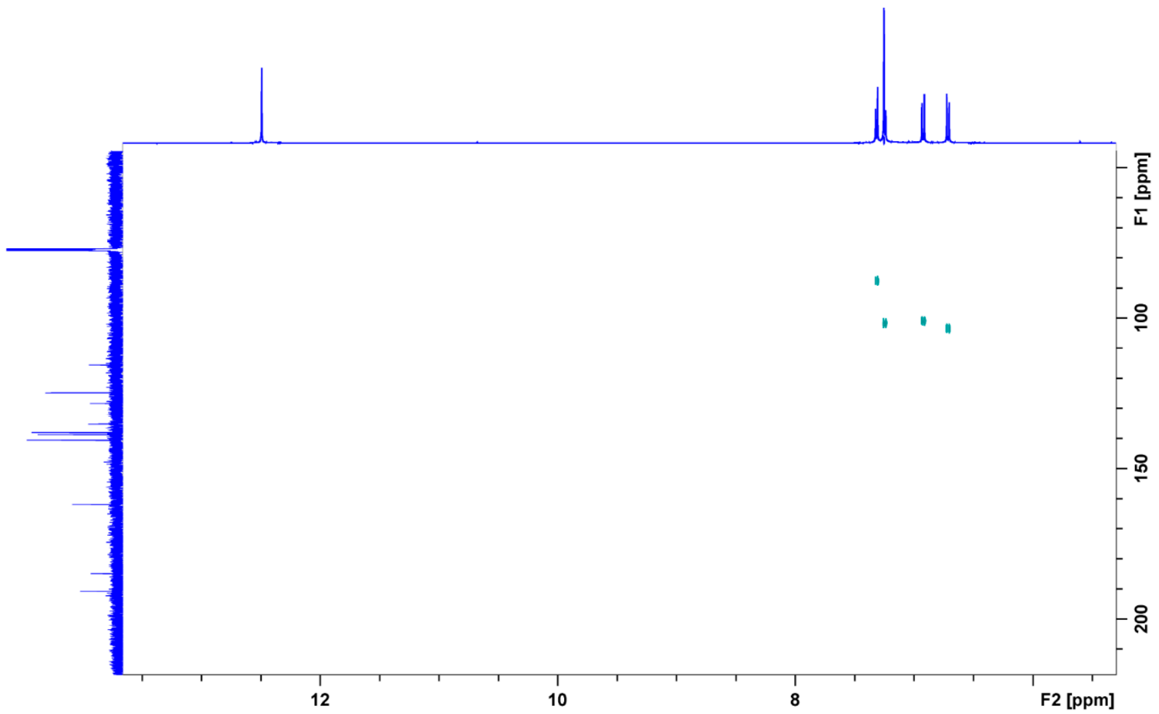


**Figure A6.** HSQC spectrum of 8,8’-bijuglone (**1**) in $CDCl_3$ (500 MHz).

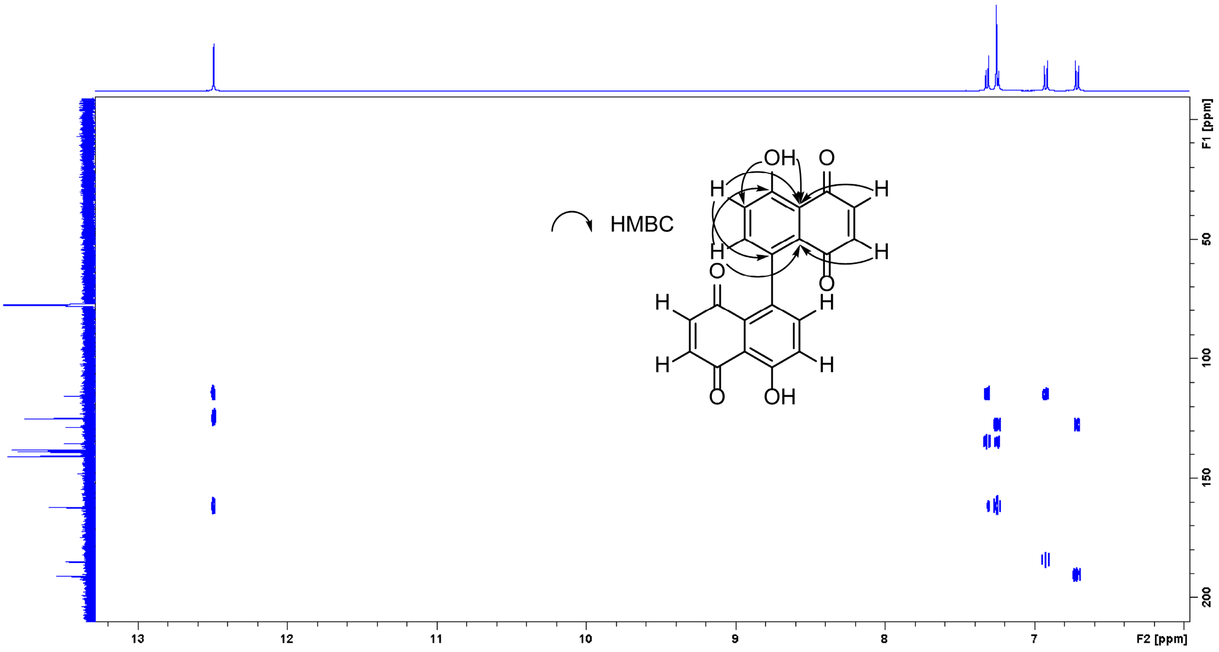


**Figure A7.** HMBC spectrum of 8,8'-bijuglone (**1**) in $CDCl_3$ (500 MHz).

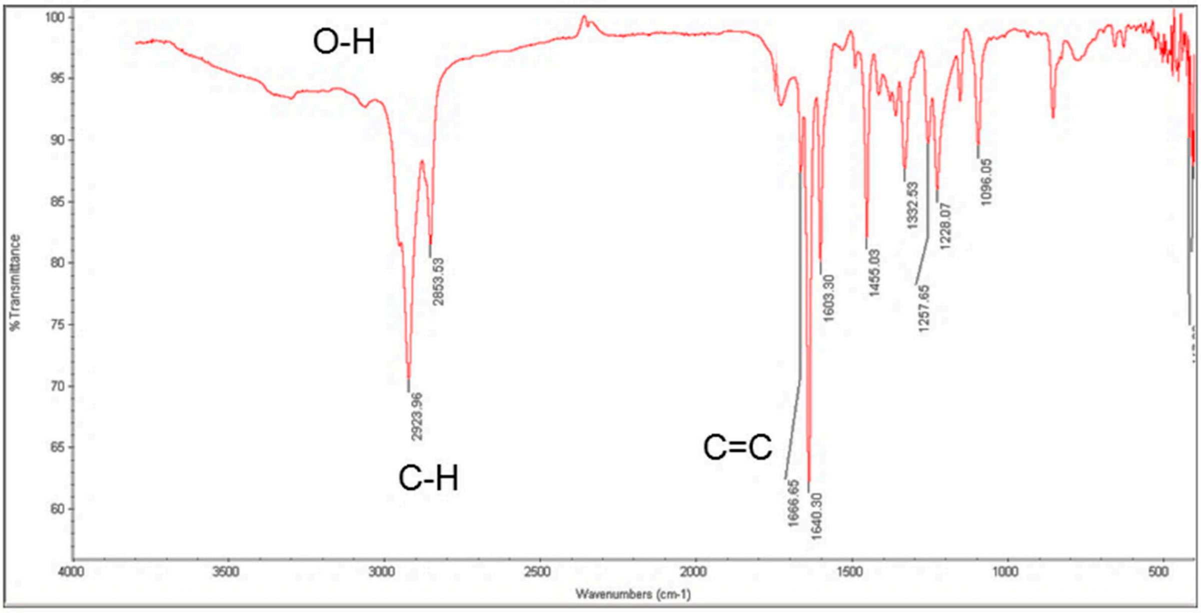


**Figure A8.** IR spectrum of 8,8’-bijuglone (**1**).
